# Ballistic and Electromagnetic Shielding Properties of Epoxy Resin Reinforced with Carbon Black and Jute Fabric

**DOI:** 10.3390/polym17030336

**Published:** 2025-01-26

**Authors:** Raphael Henrique Morais Reis, Roberto da Costa Lima, Sergio Neves Monteiro, André Ben-Hur da Silva Figueiredo, Clara Luz de Souza Santos, Lúcio Fábio Cassiano Nascimento

**Affiliations:** 1 Department of Materials Science, Military Institute of Engineering—IME, Praça General Tibúrcio 80, Urca, Rio de Janeiro 22290-270, RJ, Brazil; snevesmonteiro@gmail.com (S.N.M.); abenhur@ime.eb.br (A.B.-H.d.S.F.); claraluz@ime.eb.br (C.L.d.S.S.); lucio@ime.eb.br (L.F.C.N.); 2 Materials Group, Chemical Technology Division, Navy Research Institute (IPQM), Ipiru 2 St, Ilha do Governador, Rio de Janeiro 21931-095, RJ, Brazil; costalima.roberto@marinha.mil.br

**Keywords:** carbon black, epoxy matrix, jute fabric reinforcement, tensile properties, ballistic test, electromagnetic shielding effectiveness

## Abstract

This study explores the development of a multifunctional composite material by incorporating carbon black (CB) into an epoxy matrix reinforced with 30 vol.% jute fabric. The objective was to evaluate the impact of CB on the composite’s tensile properties, ballistic performance, and electromagnetic shielding effectiveness (SE) within the X-band frequency range (8.2–12.4 GHz). The epoxy composite with 30 vol.% jute and 5 vol.% CB (EJ30/CB5) exhibited 15% improvements in its tensile strength and elastic modulus compared to the epoxy composite with 30 vol.% jute (EJ30) only. Ballistics tests indicated no significant increases in absorbed energy or limit velocity, which may be attributed to the structural rigidity introduced by the CB. An electromagnetic shielding analysis revealed that the CB addition significantly enhanced the SE from ~2 dB in neat epoxy to 5–8 dB in EJ30/CB5, with absorption emerging as the primary shielding mechanism. The findings highlight the potential of CB- and jute-reinforced epoxy composites for applications requiring both mechanical robustness and electromagnetic interference shielding.

## 1. Introduction

The increasing demand for sustainable and high-performance materials in engineering has led to extensive research into polymer-based composites, particularly those utilizing epoxy resins due to their adaptability across the aerospace, automotive, and defense sectors [1,2]. With a growing focus on environmental sustainability, natural fiber reinforcements such as jute have gained attention for their cost-effectiveness and eco-friendly nature [2]. Jute, in particular, offers a unique combination of favorable mechanical properties, such as high tensile strength and durability, while simultaneously reducing the environmental impact compared to synthetic fibers. This makes jute a valuable reinforcement material as it can enhance the overall performance of composites without significantly increasing costs, thus positioning it as a promising alternative in various industrial applications [3,4,5].

A significant avenue of development is in multilayer armor systems (MAS), where a multi-material approach contributes to ballistic resistance, with potential integration for electromagnetic interference (EMI) shielding, which is especially pertinent in the design of ballistic vests and MAS [6,7].

Recent advances in defense technology underscore the relevance of such materials, particularly in countering threats posed by directed energy weapons (DEWs). Military technologies such as the active denial system (ADS), which rely on concentrated electromagnetic energy to produce disabling heat sensations upon targets, highlight the need for composites with strong electromagnetic attenuation properties to protect personnel from both kinetic and non-kinetic threats [8]. These “future soldiers” will likely require multifunctional protective gear that integrates ballistic resistance with EMI shielding, a combination that provides protection against both physical projectiles and DEW sources [9].

Carbon black (CB) has emerged as a potent addition agent in these contexts, as its inclusion in composites enhances both their conductivity and mechanical strength, which are key factors in achieving superior EMI shielding effectiveness (SE). Due to its electrical conductivity property, a 5 vol.% of CB loading can reach the percolation threshold, enabling a conductive network that acts as a barrier to electromagnetic waves [10]. According to Leão et al. [11], clean polyvinylidene fluoride scrap (rPVDF) composites with 2.7 vol.% CB achieved similar AC conductivity to those with 8.3 vol.% expanded graphite, underscoring CB’s superior efficiency as a conductive filler. Research has demonstrated that incorporating CB into various polymer matrices substantially enhances their SE. For instance, in an epoxy matrix, the absorbance increased from 28% at a 5 wt.% CB loading to 67% at a 20 wt.% CB loading, reaching the percolation threshold, after which it decreased to 58% at a 25 wt.% CB loading. Across the 0.1–0.13 GHz frequency range, higher CB concentrations have been shown to improve conductivity, which, in turn, boosted absorbance, decreased transmittance, and increased reflectance, achieving up to 22% SE in the C and X bands (4–10 GHz) and 47% absorbance in the same frequency range [12,13].

Recent studies have underscored the effectiveness of modified natural fiber composites in enhancing electromagnetic shielding, particularly when combined with conductive coatings. In one study, hemp fiber coated with electroless nickel-phosphorus was integrated into carbon fiber epoxy composites, yielding significant EMI shielding improvements across the X-band (8–12 GHz). With one layer of coated hemp fiber, the SE increased from 61.17 dB to 83.16 dB, and a second layer further elevated the SE to 92.77 dB—an increase of 51.66% compared to the uncoated composite [14]. Additionally, research on kenaf fiber-reinforced high-density polyethylene composites with and without multi-walled carbon nanotubes (MWCNTs) demonstrated SE values above 30 dB in the X-band at a 16% fiber content and 5 wt.% MWCNTs, with enhancements in mechanical properties, making them suitable for applications like electronic casings [15]. Moreover, ramie fiber coated with electroless nickel-phosphorus and graphene achieved an SE increase from 47.12 dB to 51 dB in the X-band and a 74% rise in conductivity, emphasizing its viability for EMI shielding and energy storage [16].

The increasing prevalence of electromagnetic interference (EMI) has prompted significant research into materials that can effectively shield against electromagnetic radiation. One promising approach involves the use of epoxy matrix composites reinforced with carbon black (CB), which combine the mechanical properties of the epoxy with the conductive capabilities of CB. These composites have been considered for a wide range of applications, including antistatic charge dissipation, EMI shielding, and stealth technology.

Aal et al. [17] highlighted the potential of epoxy–CB composites for electromagnetic shielding, demonstrating that the microstructures and mechanical properties of these composites improved with the incorporation of CB. The study showed that the interaction between the CB particles and the epoxy resin enhanced the composite’s mechanical behavior, which was superior to that of a green epoxy. Additionally, the electromagnetic shielding properties of the composites were influenced by both reflection and absorption mechanisms, with the shielding effectiveness being dependent on the volume fraction of the CB and the frequency of the electromagnetic waves. The study concluded that these composites were effective in applications requiring EMI shielding and antistatic charge dissipation.

A further investigation by Fallah et al. [18] explored the shielding effectiveness of polymer nanocomposites containing CB nanoparticles (CBN) and magnetite nanoparticles. The study revealed that shielding efficiency increased with the thickness of the nanocomposite, with a maximum shielding efficiency of 36.6 dB at 8.2 GHz for composites with 50% CBN and 15% magnetite. This research emphasized the importance of both electrical and magnetic losses in enhancing the shielding effectiveness of epoxy-based composites. The study suggested that the proper combination of nanoparticles could improve shielding in low-thickness composites.

Vovchenko et al. [19] focused on the use of graphite fillers, including ultra-dispersed graphite and graphene nanoplatelets (GNPs), for EMI shielding in epoxy-based composites. The results showed that composites with GNPs exhibited superior shielding efficiency compared to those with conventional graphite fillers. The research demonstrated that the smaller size and better electrical characteristics of GNPs contributed to a lower percolation threshold and higher EMI shielding capability. For a 10 wt.% GNP–epoxy composite, the shielding efficiency reached 8–10 dB. This research demonstrated that GNPs were highly effective in EMI shielding, with potential for thin-film applications.

Banerjee et al. [20] provided a comprehensive review on the development of epoxy-based composites for EMI shielding. The study emphasized the importance of carbonaceous reinforcements, such as CB particles and fiber mats, in enhancing the shielding effectiveness of epoxy composites. The review also discussed the synthesis of hybrid nanoparticles within an epoxy matrix, which combined both reflection and absorption losses to provide superior shielding. The study concluded that epoxy composites, particularly those reinforced with carbon-based materials, were a promising alternative to conventional metals for EMI shielding due to their lightweight structure, mechanical properties, and effectiveness in both structural and shielding applications.

The integration of carbon black into epoxy matrix composites demonstrates great potential for EMI shielding applications. The literature indicates that the mechanical and electromagnetic properties of these composites can be finely tuned by adjusting the concentration of carbon black, adding other nanoparticles and natural fibers, and controlling the composite thickness.

This study sought to expand on these findings by examining the impact of CB within a jute-reinforced epoxy matrix, a composite anticipated to perform well in both ballistic and electromagnetic protection. Statistical analyses, such as ANOVA and Weibull, were applied to evaluate the variations in tensile strength, absorbed energy, ballistic limit velocity, and SE between the CB-modified and unmodified matrices, providing insight into CB’s role in enhancing multifunctional protective properties. This research underscores the potential of CB-enhanced composites as a sustainable, effective solution for advanced protective applications, and it is particularly relevant for personal protective equipment designed for military and law enforcement use.

## 2. Materials and Methods

### 2.1. Composite Processing

The composites were fabricated using a bisphenol-A diglycidyl ether (DGEBA) epoxy resin and a triethylenetetramine (TETA) catalyst (Epoxyfiber, Rio de Janeiro, Brazil). For reinforcement, plain-weave jute fabric with an a real density of 330 g/m^2^ was purchased from Sisal Sul (Brazil), and CABOT Vulcan XC72 carbon black was also purchased from Sisal Sul. The jute fabric was pre-dried at 80 °C for 24 h in a Quimis Q317M-22 oven.

After the moisture removal from the jute fabric, the epoxy resin, TETA catalyst, and CB were manually mixed using a stoichiometric resin/catalyst ratio of 13:1. The composite molding was performed by hydraulic compression using lubricated steel molds to facilitate demolding. The composite was prepared by adding alternating layers of the DGEBA/TETA/CB mixture and jute fabric. First, the resin was added, followed by the jute fabric, and the addition of resin and fabric was alternated until the desired ratio was reached. The components of the composite were added in such a way that the resin fully wet the fabric, promoting interfacial adhesion. A semi-automatic hydraulic press (NOWAK model) applied an initial load of 3 tons for 1 min, followed by 5 tons for 24 h. For electromagnetic and other analyses, the samples were designated as follows: neat epoxy, epoxy composite with 30 vol.% jute (EJ30), epoxy composite with 5 vol.% CB (E/CB5), and epoxy composite with 30 vol.% jute and 5 vol.% CB (EJ30/CB5), maintaining consistent nomenclature across all analyses. The choice of using only a 5 vol.% of CB was made based on economic feasibility, aiming to significantly reduce the cost of the composite by increasing the percentage of jute fabric. Additionally, a higher content could have led to particle agglomeration, which could have caused defects in the samples and impaired their properties.

Due to experimental challenges, such as material shortages in certain groups, not all tests included all sample groups. The composition of each composite group and the tests they were subjected to are presented in Table 1. Not all sample types were included in each comparative study.

### 2.2. Characterization

#### 2.2.1. Scanning Electron Microscopy (SEM)

A morphological analysis was conducted via scanning electron microscopy (SEM) using an FEI Quanta 250 FEG-SEM (Hills-Boron, Boron, CA, USA) at the Electron Microscopy Laboratory (LME/IME). The surface morphologies of the EJ30 and EJ30CB5 composites were examined along their transverse axes after cryogenic fracture. Specimens were mounted on carbon tape stubs and coated with a thin gold layer prior to imaging.

#### 2.2.2. Tensile Testing

**Figure 1:**
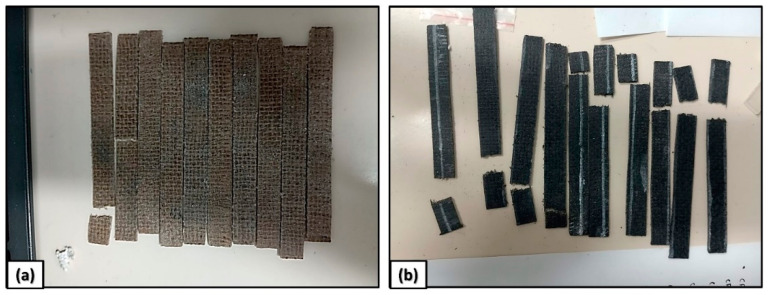
Samples used in the tensile test of the epoxy/jute/CB composites: (**a**) EJ30; and (**b**) EJ30/CB5.

The tensile tests followed the ASTM D3039/D3039M17 [16] standards at a non-destructive testing laboratory (LNDC/UFRJ) using an INSTRON 2310 testing machine (Rio de Janeiro, Brazil) with a 10 kN load cell and a crosshead speed of 2 mm/min. The EJ30 and EJ30CB5 samples were prepared to the following dimensions: 12 mm width, 120 mm length, and 2 mm thickness. Images of the specimens used in the tensile test are shown in Figure 1.

#### 2.2.3. Absorbed Energy and Ballistic Limit Velocity

Ballistics tests were conducted at a ballistic testing laboratory (LEB/IME, Rio de Janeiro, Brazil) using an Airforce Texan air rifle and 0.45 caliber lead projectiles (14.4 g). Two Pro Chrono ballistic chronographs were placed 10 cm before and after the target to record impact and residual velocities. The rifle was positioned 5 m from the target, with frontal impact on the composite plates. The absorbed energy *E_abs_* was calculated as follows: $E_{abs}=\frac{m_{p}(v_{i}^{2}-v_{r}^{2})}{2}$, where $m_{p}$ is the projectile mass, $v_{i}$ is the impact velocity, and $v_{r}$ is the residual velocity. The ballistic limit velocity, $V_{L}$, was calculated as follows: $V_{L}=\sqrt{\frac{2E_{abs}}{m_{p}}}$ [21,22].

#### 2.2.4. Shielding Effectiveness (SE)

The electromagnetic shielding effectiveness (SE) was evaluated in collaboration with the Navy Research Institute (IPqM, Rio de Janeiro, Brazil) using an N5232A PNA-L Network Analyzer over the 8.2–12.4 GHz frequency range (X-band). Samples were prepared as rectangular waveguides (10.16 mm × 22.86 mm, 7 mm thickness). The SE was calculated from scattering parameters (S-parameters) with the following expression: ${SE}_{overall}={SE}_{A}+{SE}_{R}+{SE}_{MR}$, where A is the absorption, R is the reflection, and MR refers to multiple reflections [11,12,13,14,15]. The shielding efficiency was obtained considering the calculation theory, with Equations (1) and (2) being equivalent to the relationship between the S parameter and ${SE}_{A}$ and ${SE}_{R}$, respectively [23], as follows:

(1) $${SE}_{A}=10{log}_{10}\left(\frac{1}{1-{\left|S_{11}\right|}^{2}}\right),\;\mathrm{and}$$

(2) $${SE}_{R}=10{log}_{10}\left(\frac{1-{\left|S_{11}\right|}^{2}}{{\left|S_{21}\right|}^{2}}\right).$$

In certain cases, multiple reflections can be disregarded as a shielding mechanism. This occurs, for instance, when the material thickness exceeds the skin depth or when the absorption loss (SEA) is greater than 10 dB. In these scenarios, the effect of multiple reflections is negligible (SEM ≈ 0) [16]. For the composite under investigation, with a thickness of 7 mm, and based on prior studies on penetration depth for similar materials, the contribution of the shielding mechanism by multiple reflections was disregarded [15,23,24,25].

## 3. Results and Discussion

### 3.1. Morphological Analysis

**Figure 2:**
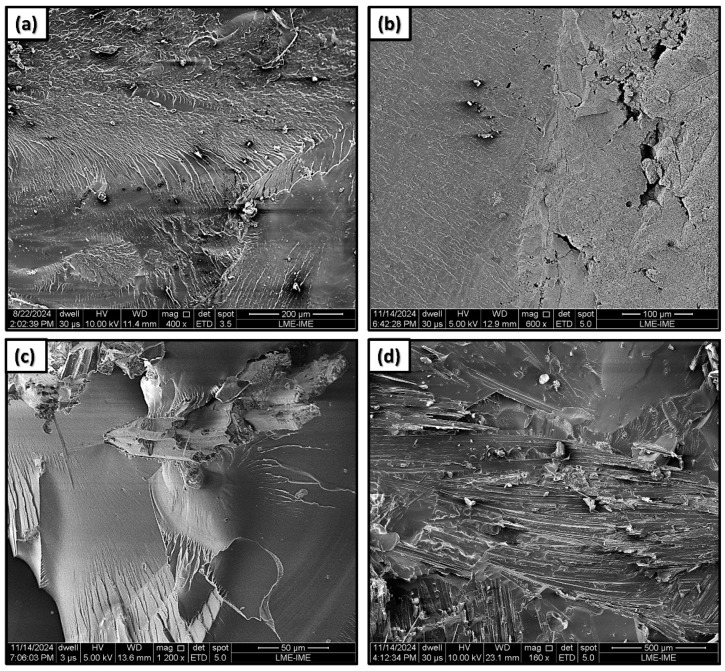
SEM micrographs of the composites: (**a**) epoxy; (**b**) E/CB5; (**c**) EJ30; and (**d**) EJ30/CB5.

Figure 2 presents the fracture surface of the epoxy/jute/CB composites analyzed by SEM. The morphological analysis of the epoxy composites reinforced with jute fabric and filled with CB revealed significant structural features that may have directly impacted the material’s mechanical and SE properties.

In Figure 2a, the fracture surface of the epoxy can be observed, where fracture mechanisms characterized by “river marks” are identified. The incorporation of CB (carbon black) into the epoxy matrix resulted in significant morphological changes, as illustrated in Figure 2b for the E/CB5 composite. In this case, the presence of “river marks” was considerably reduced, and the composite exhibited a visually “crumblier” texture. Due to the small size of the CB particles, they were not visible at low magnifications.

Figure 2c shows the micrograph of the EJ30 composite, revealing a surface characteristic of composites reinforced with natural fibers. The interaction between the matrix and the fibers is evident, with adequate adhesion between them. The epoxy matrix exhibited river marks” that reflected the directional pattern of the jute fabric, as well as evidence of the fiber pull-out and breakage caused by the cryogenic fracture used for the analysis.

Figure 2d, in turn, reveals the morphology of the EJ30/CB5 composite, highlighting the presence of CB particles that formed agglomerates dispersed throughout the epoxy matrix. These agglomerates were clearly visible, suggesting a specific interaction between the CB, the matrix, and the fibers. The small particle size of the CB may have led to a high surface energy, which facilitated the agglomeration of the material throughout the composite, as observed in Figure 2b. These structures may have been associated with the formation of conductive networks due to the presence of the CB in the polymer matrix, creating pathways for electrical conduction [25].

The morphological characteristics shown in Figure 2c were consistent with other micrographs of CB dispersed in a polymer matrix [25,26,27]. The electrical conduction model of tunneling between CB particles, as described by Balberg [28], suggests that the proximity between particles generates variable resistance within the material, depending on the distribution of interparticle distances. This behavior was reflected in the observed structure, where regions concentrated with CB alternated with matrix areas without CB, resulting in a non-homogeneous conduction system.

### 3.2. Tensile Results

Table 2 presents the results for the tensile strength and elastic modulus obtained from the tensile test.

The results shown in Table 2 illustrate a comparison between the composites, highlighting the influence of the CB. It was observed that the addition of CB improved both the tensile strength and elastic modulus. The EJ30 composite exhibited a tensile strength of 37.51 MPa, while the EJ30/CB5 reached 43.18 MPa, representing an increase of approximately 15%. This improvement was attributed to the additional reinforcement provided by the CB, which enhanced the load transfer between the matrix and the fibers, and it reduced micro cracks. The elastic modulus also increased, rising from 4262.69 MPa (EJ30) to 4927.33 MPa (EJ30/CB5), an increment of approximately15.6%. This occurred due to the rigidity of the CB particles, which reinforced the matrix and increased the resistance to elongation.

Previous studies have demonstrated that the mechanical properties of an epoxy resin can be significantly enhanced through reinforcement with natural fibers. According to Zolfakkar et al. [29], natural fibers contribute to a notable improvement in both tensile strength and the modulus of elasticity. As shown by Sathiyamoorthy et al. [30], CB exhibited a comparable effect in enhancing the mechanical properties of the jute-reinforced composites investigated in this study. The incorporation of 5 vol.% CB in the epoxy matrix led to notable improvements in the mechanical properties of the composites. As shown in Table 2, the tensile strength and elastic modulus increased by 15% for the EJ30/CB5 composite compared to the composite EJ30.

A statistical analysis of the tensile test results for the composites was performed using Weibull and ANOVA tests. The mechanical properties evaluated, including tensile strength, elastic modulus, and elongation, were analyzed in detail. The parameters obtained from the Weibull analysis are presented in Table 3.

The results showed that the coefficient of determination (R^2^) exceeded 0.9, indicating an excellent fit of the data to the statistical model. The scale parameter (θ) revealed that in 63.2% of the cases, the elastic modulus and tensile strength exceeded their mean values. Furthermore, the shape parameter (β) was greater than one for all the properties and samples evaluated. This β value suggested that the θ values shown in Table 3 were associated with an increasing failure rate as the number of tested samples increased [31,32].

The ANOVA analysis was conducted to evaluate whether the differences in the tensile strength and elastic modulus values between the EJ30 and EJ30/CB5 composites were statistically significant. The parameters obtained for tensile strength are summarized in Table 4.

The ANOVA analysis was conducted to evaluate whether the differences in the tensile strength and elastic modulus values between EJ30 and EJ30/CB5 were statistically significant. For the tensile strength, the F-value of 1.939 and the *p*-value of 0.179 indicated that the difference between treatments was not statistically significant at the commonly used significance level of 0.05. This suggested that the incorporation of CB did not result in a significant change in tensile strength under the tested configurations. The high residual sum of squares, as compared to the sum of squares for the treatments, highlighted considerable variability in the data, which may have contributed to the lack of statistical significance. For the elastic modulus, the F-value of 4.252 and the *p*-value of 0.058 approached the threshold for statistical significance but did not conclusively confirm a significant difference at the 5% level. However, this result indicated a trend toward significance, suggesting that the addition of carbon black may have had a measurable effect on the elastic modulus, which could have become statistically significant with additional replicates or reduced data variability. The proportion of variability explained by the treatments was slightly higher for the elastic modulus compared to the tensile strength, further reinforcing the possibility of a subtle influence of CB on this property. These findings suggested that the inclusion of carbon black in the EJ30 matrix did not lead to statistically significant changes in tensile strength or elastic modulus under the tested conditions.

**Figure 3:**
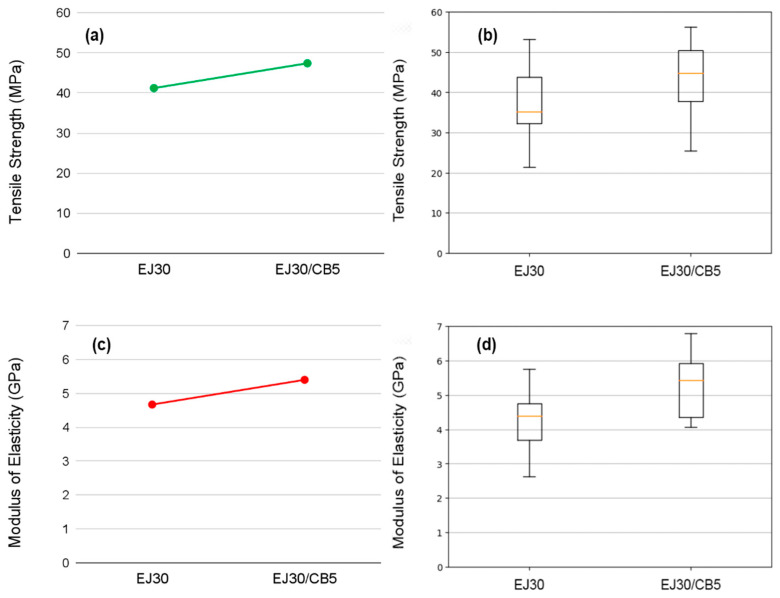
Comparative visualization of the average values, with tensile strength (**a**) and elastic modulus (**c**) for the EJ30 and EJ30/CB5 samples, alongside box-and-whisker plots for tensile strength (**b**) and elastic modulus (**d**) for the EJ30 and EJ30/CB5 composites.

Figure 3 visually illustrates the ANOVA results. Figure 3a,c displays the mean tensile strength and elastic modulus for the neat EJ30 and EJ30/CB5 composites. The addition of jute fabric reinforcement (EJ30) enhanced both properties compared to the neat epoxy [29]. Furthermore, the incorporation of CB (EJ30/CB5) slightly improved these values compared to the EJ30 alone. The box-and-whisker plots in Figure 3b,d depict a wide but comparable data distribution for tensile strength across the EJ30 and EJ30/CB5 composites, consistent with the *p*-value of 0.179. For the elastic modulus, the broader data dispersion observed in Figure 3d aligned with the *p*-value of 0.058, indicating a somewhat more variable response for this property.

### 3.3. Ballistic Test Results

**Figure 4:**
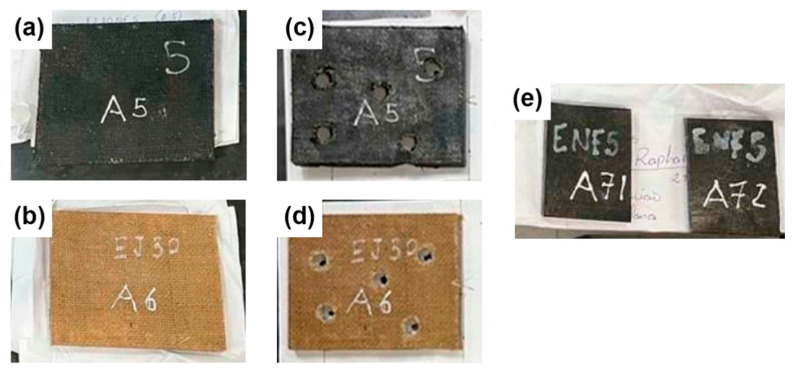
Images of EJ30/CB5 (**a**) and EJ30 (**b**) before impact, EJ30/CB5 (**c**) EJ30/CB5 after impact, EJ30 (**d**) EJ30 after impact, and (**e**) E/CB5 before impact.

Figure 4 shows the EJ30 and EJ30/CB5 samples before and after the ballistics tests (Figure 4a,c for EJ30/CB5; Figure 4b,d for EJ30; and Figure 4e for E/CB5). The samples showed adequate spacing between impact sites, and the addition with CB produced a color change from beige (EJ30) to black (EJ30/CB5). Similarly, the E/CB5 samples exhibited a color change to black following the addition of carbon black, consistent with the EJ30/CB5 samples. However, the E/CB5 samples lacked structural integrity after ballistic impact, which was attributed to the absence of reinforcement by the jute fabric, which prevented the recovery of their fragments.

The ballistic performance results indicated the influence of the reinforcement and CB addition on the composite’s behavior under impact. The data in Table 5 show the progressive decreases in both the limit velocity (*V*_*L*_) and absorbed energy (*E*_*abs*_) with each reinforcement increment of the jute fabric.

The inclusion of jute fabric (EJ30) resulted in a 5.4% reduction in *V*_*L*_ and a 10.9% decrease in *E*_*abs*_ compared to the E/CB5 sample. This reduction could be attributed to the inherent mechanical properties of the jute fibers, which, while increasing the composite’s stiffness, may have created localized stress concentrations that reduced its ability to dissipate energy effectively during the high-velocity impacts. The decrease in *V*_*L*_ suggested a lower capacity to resist projectile penetration, while the reduction in *E*_*abs*_ reflected diminished energy absorption capabilities, likely due to the non-homogeneous energy distribution throughout the composite’s structure.

The incorporation of 5 vol.% CB in the EJ30 (EJ30/CB5) sample slightly mitigated these effects, resulting in less than 1.2% additional reductions in both *V*_*L*_ and *E*_*abs*_ compared to the EJ30 sample. Conversely, the incorporation of the same volume fraction of carbon black into the epoxy matrix resulted in approximate increases of 5.6% in *V*_*L*_ and 12.2% in *E*_*abs*_ compared to the EJ30 sample. This indicated that, although carbon black did not enhance the structural integrity of the composite, it played a significant role in improving its ballistic behaviors. The minimal further decrease between EJ30 and EJ30/CB5 indicated that the incorporation of CB did not significantly compromise the ballistic performance. This could be explained by the formation of localized conductive networks by the CB particles, which may have improved the stiffness and interfacial bonding between the matrix and the fibers, counterbalancing the adverse effects introduced by the jute fabric reinforcement.

The Weibull parameters and ANOVA results for the ballistic behavior of the EJ30 and EJ30/CB5 composites are presented in Table 5 and Table 6, respectively. As shown in Table 5, the Weibull parameters for the limit velocity (*V_L_*) and absorbed energy (*E_abs_*) indicated notable differences between the EJ30 and EJ30/CB5 composites. Both samples exhibited β (shape parameter) values greater than one, suggesting that the data followed a Weibull distribution, with a tendency for increased reliability as the analyzed property increased. Statistical analyses could not be performed for the E/CB5 samples due to the insufficient amount of data collected, limiting the robustness of conclusions for this specific condition.

However, the EJ30 sample stood out, with R^2^ values exceeding 0.9 (0.90 for *V_L_* and 0.98 for *E_abs_*), whereas the EJ30/CB5 sample showed lower R^2^ values (0.81 for *V_L_* and 0.76 for *E_abs_*). These results indicated that the Weibull model better explained the data variability of the EJ30 sample compared to the EJ30/CB5 sample. The inconsistent distribution of CB particles could have been one of the reasons for the greater data dispersion in the EJ30/CB5 sample, as it may have negatively affected the ballistic properties of this composite. The θ (scale parameter) values were similar between the composites for both properties, with minimal differences between the EJ30 and EJ30/CB5 samples. For *V_L_*, the value of θ was slightly higher in the EJ30 sample (198.00 m/s versus 197.10 m/s in the EJ30/CB5 sample). Similarly, for *E_abs_*, the EJ30 sample presented a θ value of 286.40 J, slightly higher than that of the EJ30/CB5 sample (283.60 J). This indicated that, in terms of overall capacity, the two materials had comparable performances, but with more consistent variations in the EJ30 sample due to the higher R^2^ value.

The ANOVA results presented in Table 7 indicated no statistically significant differences between the EJ30 and EJ30/CB5 samples for *V_L_* (*p* = 0.57) and *E_abs_* (*p* = 0.56), with F-values of below one in both analyses.

This implied that the addition of CB did not substantially alter the evaluated ballistic parameters. However, it is crucial to interpret these findings considering the mechanisms governing the ballistic behavior of polymer composites. The reinforcement with jute fabric and CB particles may have had potential impacts on *E_abs_* and *V_L_* due to the interaction between the matrix and the reinforced phases. For example, although the ANOVA results did not indicate significant differences, the slightly higher θ values for the EJ30 sample and the higher R^2^ suggested that the distribution and adhesion between the matrix and the jute played a more efficient role in the EJ30 sample than in the EJ30/CB5 sample. The lack of statistical significance in the ANOVA results did not completely invalidate the influence of the CB reinforcement. However, it emphasized the need to investigate other factors, such as the distribution of CB particles, the degree of interfacial adhesion, and the homogeneity of the reinforced phases, to fully understand the differences observed in the data variability (the R^2^ values). Additionally, further studies could consider additional variables, such as density and mechanical strength, to explore the combined impact of the materials used.

**Figure 5:**
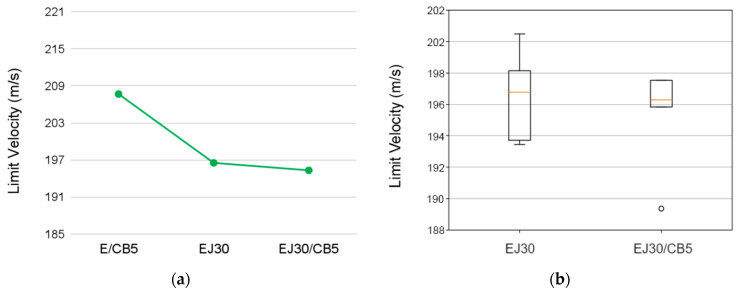
Comparison plots showing (**a**) the mean limit velocity values for the neat epoxy, EJ30, and EJ30/CB5 samples and (**b**) box-and-whisker plots for the limit velocity values of the EJ30 and EJ30/CB5 samples.

A comparison between the EJ30 and EJ30/CB5 samples (Figure 5) revealed that combining jute and CB created a hybrid matrix that, while mechanically stronger, did not lead to significant improvements in $E_{abs}$ and $V_{L}$ values [30]. This behavior aligned with the literature on polymer composites, where natural fiber reinforcements and conductive particles have impacted mechanical and thermal properties but have not necessarily enhanced energy dissipation capacities [33,34].

Natural fiber composites with conductive fillers are known for their rigidity and impact resistance compared to a pure polymer matrix like epoxy. However, the $V_{L}$ values of reinforced composites depend on the deformation ability of both their fibers and matrices, as well as their interfaces. In this case, the interfacial cohesion may have been insufficient to form a high-energy-absorbing matrix, as would be observed in composites reinforced with synthetic fibers like Kevlar, which have optimized structures and interfaces for high-speed impact absorption [35].

In polymer materials reinforced with carbon fillers, similar to CB, the ballistic impact tends to cause less significant matrix deformation, which increases rigidity but limits energy absorption [36]. This may explain the low F-value for $E_{abs}$ shown in Table 7, even with the CB addition, indicating that the conductive particles did not significantly improve the energy dissipation in this context.

**Figure 6:**
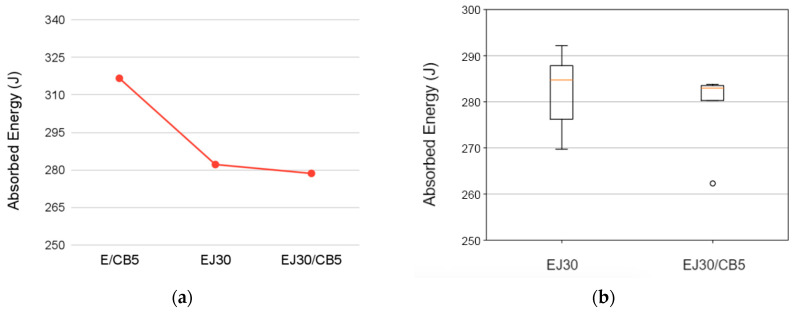
Comparison plots showing (**a**) the mean absorbed energy values for the neat epoxy, EJ30, and EJ30/CB5 samples and (**b**) box-and-whisker plots for the absorbed energy values of the EJ30 and EJ30/CB5 samples.

In the EJ30/CB5 sample, this CB filler primarily acted as a conductive and reinforcing agent, improving the composite’s mechanical strength and rigidity. CB as an additive enhances impact resistance and stiffness in composites, though its impact on energy absorption is limited as its primary function is to increase resistance rather than dissipate energy [29]. The addition of carbon black (CB) to the epoxy matrix slightly reduced structural integrity while significantly increasing absorbed energy, as is visually evident in the plot shown in Figure 6a. Conversely, when combined with jute fabric, the CB resulted in less data dispersion for the $E_{abs}$ values, as shown in Figure 6b.

**Figure 7:**
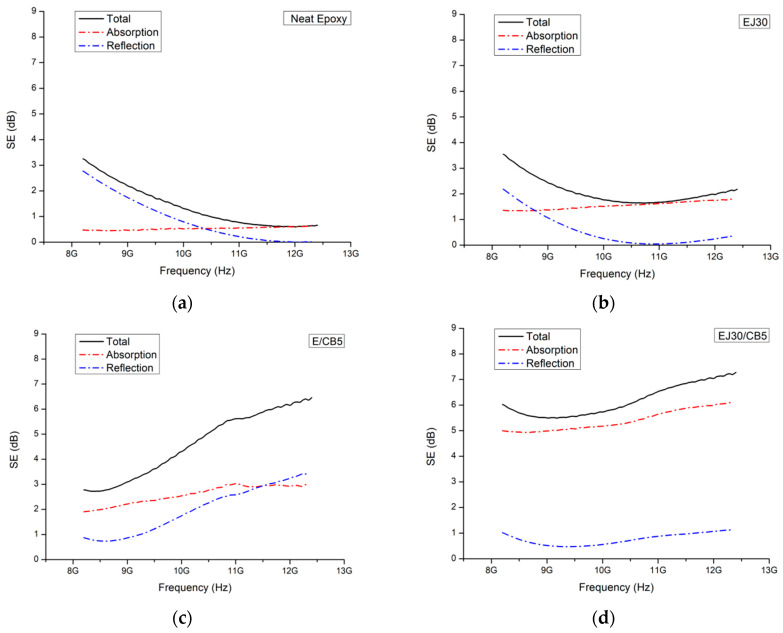
Shielding effectiveness (SE) for the (**a**) neat epoxy, (**b**) EJ30, (**c**) E/CB5, and (**d**) EJ30/CB5 samples.

The SE analysis shown in Figure 7 for the samples reinforced with jute fabric and CB highlighted the significant impact of these reinforcements on the composites’ electromagnetic behavior. The SE improved with the addition of CB, and the predominant shielding mechanism shifted from reflection to absorption. Furthermore, jute reinforcement, especially in combination with CB, stabilized the SE across the frequency range analyzed, suggesting a possible improved shielding effectiveness.

The comparison showed that the neat epoxy exhibited a low shielding effectiveness (SE of ~1–2 dB) across the frequency range, indicating a predominantly dielectric behavior with minimal absorption. The EJ30 sample showed a slight increase in SE to around 2–3 dB, suggesting that the jute fabric alone contributed little to the electromagnetic shielding but enhanced the wave dispersion. In the E/CB5 and EJ30/CB5 samples, the SE reached 5–8 dB, demonstrating that combining jute fabric with CB significantly improved the SE.

The SE increase in the E/CB5 and EJ30/CB5 samples because the CB addition aligned with past studies showing the role of conductive particles in creating current pathways within a polymer matrix and dissipating electromagnetic energy through absorption mechanisms. According to Bertolini et al. [37], composites containing CB fillers exhibited higher electrical conductivity and better electromagnetic wave absorption, resulting in higher SE values. Similarly, Mondal et al. [38] demonstrated that adding conductive fillers to a dielectric matrix enhanced absorption, as incident waves were converted to heat through current dissipation.

The SE stabilization with the jute fabric addition in the EJ30 and EJ30/CB5 samples could be attributed to the structural and dielectric properties of the natural fibers, which dispersed electromagnetic radiation across a broad frequency range. Natural fibers like jute have a fibrous structure that aids in electromagnetic wave dispersion and increases composite mechanical strength, preventing a conductive CB structure from collapsing at high frequencies. A study by Hou et al. [39] suggested that combining natural fibers with conductive fillers created a hybrid structure that provided both mechanical stability and homogeneous conductive particle distribution, enhancing absorption and minimizing SE variations with frequency.

This combination of jute fabric and CB created a synergistic effect, maintaining high SE values across the X-band frequency range. The jute fabric, as a dielectric, supported the CB particles, forming a semi-conductive structure. Larguech et al. [40] investigated the dielectric properties of a green composite made of a polylactic acid (PLA) polybutylene succinate (PBS) polymer matrix reinforced with jute fibers, showing that while the PBS enhanced crystallinity, the addition of jute increased the glass transition temperature and introduced new dielectric relaxations associated with water dipoles and interfacial polarization.

The SE value’s dependence on frequency revealed distinct behaviors between the neat and CB-containing samples. At higher frequencies (above 10 GHz), the CB-containing samples maintained relatively stable SE values, while the neat epoxy and EJ30 samples exhibited decreasing SE values with increasing frequency, which was characteristic of dielectric materials. This phenomenon was consistent with the dielectric relaxation model, where insufficient polarization at high frequencies reduces shielding capacity [40].

**Figure 8:**
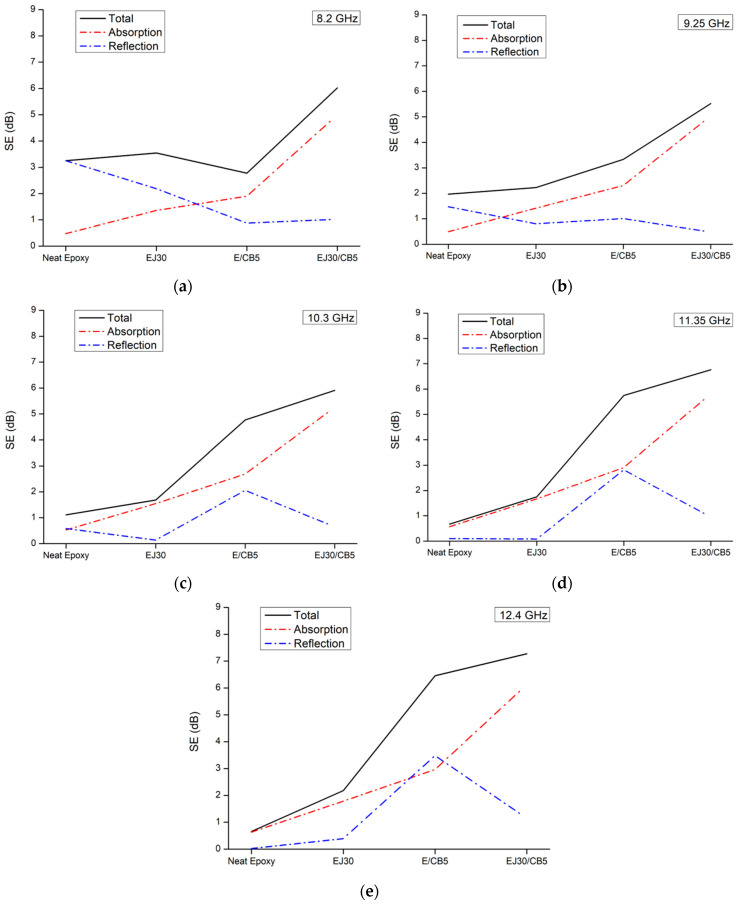
Shielding effectiveness (SE) of the samples at specific frequencies: (**a**) 8.2 GHz, (**b**) 9.25 GHz, (**c**) 10.3 GHz, (**d**) 11.35 GHz, and (**e**) 12.4 GHz.

Figure 8 shows that, for the neat epoxy and EJ30 samples, the SE shifted from reflection to absorption with the increasing frequency. This behavior aligned with polymer composite literature, where reflection is the dominant mechanism at low frequencies due to a base material’s lack of significant conductivity. As the frequency increases, the material’s polarization capacity decreases, leading to greater absorption [39]. At high frequencies, the energy of incident waves can be partially dissipated within a polymer matrix, especially in dielectric materials like epoxy.

The E/CB5 sample exhibited an unusual behavior with its shielding efficiency through reflection increasing with frequency, surpassing absorption at 12.4 GHz. This deviation from typical behavior could be attributed to the structure and distribution of the CB particles within the composite. At higher frequencies, the formation of conductive pathways within a composite may enhance the reflection of incident waves instead of allowing full absorption. According to Hou et al. [35], this phenomenon occurs when the concentration of conductive particles is sufficient to create reflective interfaces but not high enough to form a complete conductive network that would maximize absorption.

## 4. Conclusions

Incorporating 5 vol.% carbon black (CB) into a 30 vol.% jute-reinforced epoxy matrix composite effectively improved tensile properties and electromagnetic shielding without substantially altering ballistic energy absorption. This composite demonstrated enhanced shielding effectiveness primarily through absorption, achieving a more stable and effective shielding profile across a broad frequency range into the X-band. This behavior suggests that combining jute and CB within an epoxy matrix creates a hybrid structure suitable for applications where both mechanical strength and electromagnetic interference shielding are essential. These findings support the potential of natural fiber and conductive filler combinations in developing sustainable, high-performance composite materials.

## Figures and Tables

**Table 1:** Classification of the composites by composition and indications of the tests conducted for each group.

Composite	Epoxy (vol.%)	Carbon Black (vol.%)	Jute Fabric (vol.%)	Tensile Test	SEM	Ballistic Test	Shielding Effectiveness
Epoxy	100	-	-	✕	✓	✕	✓
E/CB5	95	5	-	✕	✓	✓	✓
EJ30	70	-	30	✓	✓	✓	✓
EJ30/CB5	65	5	30	✓	✓	✓	✓

**Table 2:** Tensile strength and elastic modulus results for epoxy/CB/jute composites.

Composite	Tensile Strength (MPa)	Elastic Modulus (MPa)
EJ30	37.51	4262.69
EJ30/CB5	43.18	4927.33

**Table 3:** Weibull parameters for the mechanical properties of the EJ30 and EJ30/CB5 composites.

	EJ30			E30J/CB5		
	β	θ	R^2^	β	θ	R^2^
Tensile Strength (MPa)	4.25	41.21	0.97	4.38	47.40	0.97
Elongation (%)	4.66	1.17	0.94	3.82	1.17	0.98
Elastic Modulus (MPa)	4.49	4667.15	0.97	4.50	5397.10	0.91

**Table 4:** ANOVA results for tensile strength and elastic modulus values of composites.

Property	Cause of Variation	Degrees of Freedom	Sum of Squares	Mean Square	F-Value	*p*-Value
Tensile Strength	Treatments	1	177.102	177.102	1.939	0.179
	Residual	20	1826.753	91.337		
	Total	21	2003.855			
Elastic Modulus	Treatments	1	4.118	4.118	4.252	0.058
	Residual	14	13.559	0.968		
	Total	15	17.677			

**Table 5:** Mean limit velocity and absorbed energy for the neat epoxy and EJ30 and EJ30/CB5 samples.

Materials	$V_{L}$ (m/s)	$E_{abs}$ (J)
E/CB5	207.67 ± 2.02	316.63 ± 6.78
EJ30	196.52 ± 2.99	282.14 ± 9.07
EJ30/CB5	195.31 ± 3.42	278.59 ± 9.21

**Table 6:** Weibull parameters for the EJ30 and EJ30/CB5 samples in terms of their ballistic performance regarding limit velocity and absorbed energy.

	EJ30			EJ30/CB5		
	β	θ	R^2^	β	θ	R^2^
Limit Velocity (m/s)	64.70	198.00	0.90	52.89	197.10	0.81
Absorbed Energy (J)	31.82	286.40	0.98	26.75	283.60	0.76

**Table 7:** ANOVA results for the limit velocity between the EJ30 and EJ30/CB5 samples.

Property	Cause of Variation	Degrees of Freedom	Sum of Squares	Mean Square	F-Value	*p*-Value
***V_L_***	Treatments	1	3.66	3.66	0.35	0.57
	Residual	8	82.53	10.32		
	Total	9	86.19			
***E_abs_***	Treatments	1	3.66	3.66	0.35	0.57
	Residual	8	82.53	10.32		
	Total	9	86.19			

## Data Availability

The data presented in this study are available on request from the corresponding author.
